# Identification and profile of phenolamides with anthracnose resistance potential in tea (*Camellia sinensis*)

**DOI:** 10.1093/hr/uhad154

**Published:** 2023-08-01

**Authors:** Wenzhao Wang, Xingcui Xie, Yuanyuan Lv, Haonan Guan, Lu Liu, Qian Huang, Yumeng Bao, Jie Zhou, Lu Bao, Chunmei Gong, Youben Yu

**Affiliations:** College of Horticulture, Northwest A&F University, Yangling 712100 Shaanxi, China; College of Horticulture, Northwest A&F University, Yangling 712100 Shaanxi, China; College of Tropical Crops, Hainan University, Haikou 570228 Hainan, China; College of Horticulture, Northwest A&F University, Yangling 712100 Shaanxi, China; College of Horticulture, Northwest A&F University, Yangling 712100 Shaanxi, China; College of Horticulture, Northwest A&F University, Yangling 712100 Shaanxi, China; College of Horticulture, Northwest A&F University, Yangling 712100 Shaanxi, China; College of Horticulture, Northwest A&F University, Yangling 712100 Shaanxi, China; College of Horticulture, Northwest A&F University, Yangling 712100 Shaanxi, China; College of Horticulture, Northwest A&F University, Yangling 712100 Shaanxi, China; College of Horticulture, Northwest A&F University, Yangling 712100 Shaanxi, China

## Abstract

Tea anthracnose is a prevalent disease in China that can lead to reduced tea production and lower quality, yet there is currently a lack of effective means for controlling this disease. In this study, we identified 46 phenolamides (including 27 isomers) in different tissues and organs of tea plants based on a developed workflow, and the secondary mass spectra of all these compounds have been documented. It was revealed that tea plants predominantly accumulate protonated aliphatic phenolamides, rather than aromatic phenolamides. The profile of phenolamides indicate that their buildup in tea plants is specific to certain tissues and acyl-acceptors, and this distribution is associated with the extent of phenolamide acyl-modification. Additionally, it was observed that *N*-Feruloylputrescine (Fer-Put, a type of phenolamides) was responsive to the stimulated accumulation of the tea anthracnose pathogen. The findings of anti-anthracnose experiments *in vitro* and on tea leaf demonstrated that Fer-Put was capable of significantly inhibiting the growth of anthracnose pathogen colony, effectively prevented tea leaf disease. Furthermore, it was observed that Fer-Put treatment can enhance the antioxidant enzyme activity of tea leaves. *TEA002780.1* and *TEA013165.1* gene may be responsible for the biosynthesis of Fer-Put in the disease resistance process in tea plants. Through these studies, the types and distribution of phenolamides in tea plants have been elucidated, and Fer-Put's ability to resist anthracnose has been established, providing new insights into the resistance of tea anthracnose.

## Introduction

Anthracnose is a widespread ailment in tea plantations across China, capable of inducing leaf withering and scorching, thereby causing substantial leaf loss, hindering tea plant growth and yield, and diminishing tea quality. As such, it is the most significant disease affecting tea plants in China. Despite extensive research, there is still a lack of effective chemical pesticides to control tea anthracnose, and the interaction between tea plants and pathogenic bacteria requires further investigation [1]. The exploration of the pathogenicity and resistance of tea plants holds great importance in the development of disease-resistant cultivars and the implementation of accurate disease management strategies. Thus, understanding how plants resist pathogen is an essential matter in both fundamental research and industrial application. Despite the abundance of secondary metabolites in tea plants, such as flavonoids, caffeine, and theanine, research on other secondary metabolites, particularly those with disease-resistant properties, has been relatively limited. Recent studies suggest that phenolamides, a class of plant endogenous secondary metabolite, may play an important role in plant disease resistance [2].

**Figure 1:**
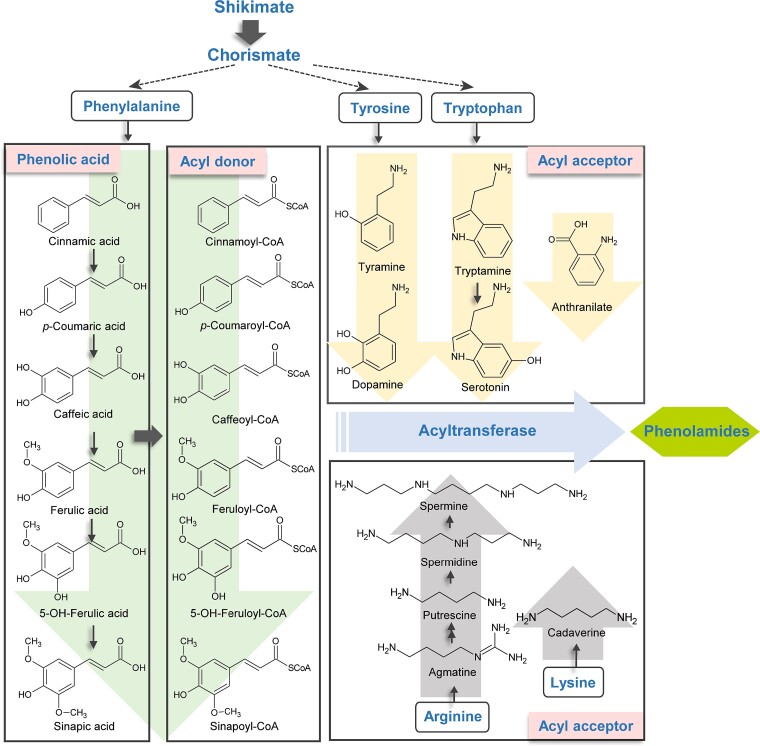
Schematic diagram of the structure of acyl donors and acceptors of phenolamides in plants.

The chemical composition of phenolamides involves acyl donor in the form of a hydroxycinnamic acid moiety and acyl receptor in the form of aliphatic or aromatic amine moiety, which are joined by an amide bond (-*CO*-*NH*-). Plants commonly contain at least six types of hydroxycinnamic acid and its derivatives, as well as five types of linear polyamines and five types of aromatic polyamines (Fig. 1). The vast array of possible phenolamides, which exceeds 5000 in theory, arises from the varied phenolic acyl groups and modified sites within polyamines [2]. Phenolamides have been identified in numerous plant species, such as barley [3], tobacco [4], tomato [5], potato [6], and sunflower [7]. The targeted metabolomic analysis approach employed by Dong *et al*. allowed for the identification of 11 distinct single acylation modified phenolamides, four secondary acylation modified phenolamides, and one tertiary acylation modified phenolamide in rice. Among these, *N*-feruloylputrescine, *N-p*-coumaroylputrescine, and *N′,N′′,N′′′-*diferuloylsinapoylspermidine were found to be predominantly present in the roots, while *N′,N′′-*disinapoylspermidine was primarily accumulated in the leaves and seed [8]. In *Arabidopsis thaliana*, two primary spermidine binding compounds have been identified in flowers and seeds as well: *N′,N′′,N′′′-*diferuloylsinapoylspermidine and *N′,N′′*-disinapoylspermidine [9, 10]. However, the current research on phenolamides in tea plants is rather limited. The metabolomic analysis by Chen *et al*. revealed that the flowers of the *‘Tieguanyin’* variety contained two phenolamides, di-*p*-coumaroylputrescine and tri-*p*-coumaroylspermidine [11]. Nonetheless, the specific types and accumulation profile of phenolamides, along with their associated physiological roles and the biosynthesis in tea plants, remain largely unexplored.

Numerous studies have demonstrated the crucial role of phenolamides in plant disease resistance. The first report of significant accumulation of phenolamides in wheat leaves infected with rust dates back to the 1970s [12]. Since then, researchers have discovered that many phenolamides can participate in the disease resistance process in various plant families, including solanaceae, cruciferae, and gramineae [13–15]. For instance, hordatines in barley has been found to effectively inhibit fungal spore germination [16], while *p*-coumaroyl-hydroxyagmatine exhibits strong resistance to powdery mildew in and out of barley [17]. Oat-specific phenolamide- avenanthramides can fully integrate into the plant cell wall when bacteria infect plants and play a disease-resistant role [18]. The biosynthesis of phenolamides in plants is completed through the action of acyltransferases, which catalyze the final step. Recently, two acyltransferase gene clusters have been identified in rice, which can synthesize hydroxycinnamoyl-tyramine and hydroxycinnamoyl-putrescine. Overexpression of biosynthetic genes involved in the gene clusters can enhance the immune response of rice, improve its resistance to bacterial blight and rice blast, and affect the process of cell apoptosis [19, 20]. It appears that phenolamides in tea plants might have similar biological effects related to disease resistance, with acyltransferases potentially being involved.

In the present research, we utilized UHPLC-Orbitrap-MS/MS and UPLC-QQQ-MS/MS to perform the identification and quantification of phenolamides in tea plants. Our findings indicate that the accumulation of these compounds is tissue-specific, and that the accumulation of *N*-Feruloylputrescine (Fer-Put, a type of phenolamides) can be induced by the presence of the tea anthracnose pathogen. The results of Oxford Cup anti-pathogen experiments *in vitro* and resistance tests on tea leaves further confirms the biological activity of Fer-Put against tea anthracnose. Moreover, it was observed that Fer-Put treatment can enhance the antioxidant enzyme activity of tea leaves, and *TEA002780.1* and *TEA013165.1* gene may be implicated in the accumulation of Fer-Put in the disease resistance process in tea plants. These findings contribute to our understanding of the mechanisms underlying anthracnose resistance in tea plants, and provide valuable insights and guidance for the development of effective plant protection strategies in practical production settings. Additionally, the research provides a foundation for the development of resistant tea varieties and for the acceleration of breeding cycles.

## Results

### Identification of phenolamides

To attain the most comprehensive mass spectrum signals of phenolamides, specimens from annual *C. sinensis* ‘*Shaancha 1*’ cutting seedlings were collected and freeze-dried, comprising of first leaf, second leaf, third leaf, fourth leaf, tender stem, and flower. To acquire more signals, UHPLC-Orbitrap-MS/MS was employed in positive ion mode to detect the mixed samples in the mass-to-charge (*m/z*) ranges of 50–200, 200–500, 500–800, 800–1000, and 1000–1500. As results, a total of 5151 primary and 31 284 secondary MS level signals were acquired (Table S1, see online supplementary material).

**Figure 2:**
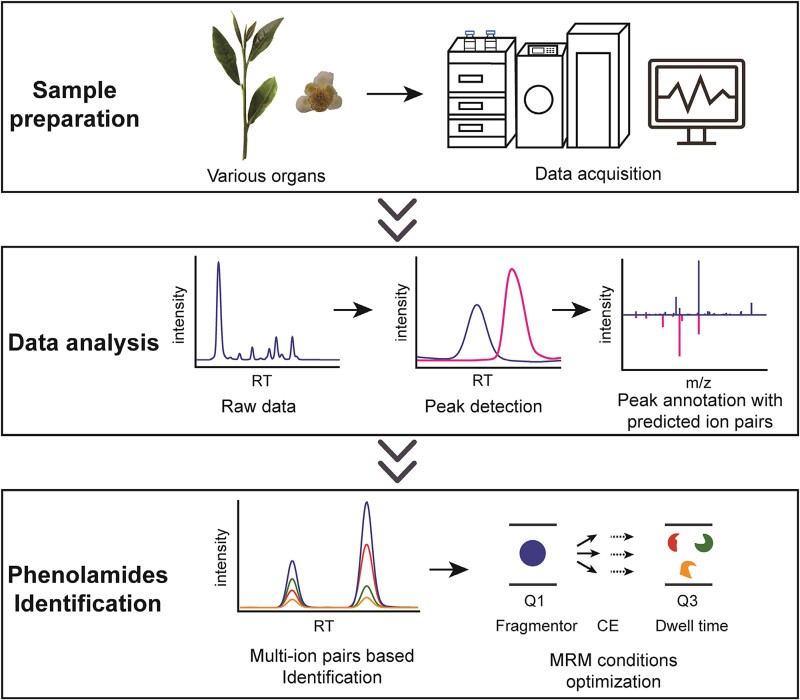
The workflow of the identification phenolamides in tea.

Because the commercial standards for phenolamide were limited, two (*N*-Feruloylputrescine and *N*-Coumaroyltyramine) were purchased to analyse the MS behaviour. First, in full scan mode, we acquired the molecular mass and retention time (RT) of the two standards. Consequently, product ion scan mode was employed to identify the product fragments and the cleavage pattern at different collision energies (0, 10, 20, 40 eV), providing the illustrations for more phenolamide derivatives which have similar structures and cleavage pattern (Fig. S1, see online supplementary material). In the total ion chromatogram, we compared the retention times, accurate protonated molecule [M + H]^+^, and cleavage patterns of candidate signals to those of the authentic standards or reported in published studies. Our research revealed that the predominant ion fragments of phenolamides were generated from the breaking of the *C*-*N* bond (Fig. S1A and F, see online supplementary material). Most aliphatic phenolamides had a characteristic ion fragment at *m/z* 72.08, which was attributable to the polyamine moiety (Fig. S1A–E, see online supplementary material). In addition, the cleavage of phenolic acyl group can produce a series of regular fragmentations. As an illustration, the molecular mass of feruloyl group was 177 at *m/z* after the detachment from feruloyl acylated phenolamide, then it decreased by CH_3_ and OH to become a *m/z* 145 fragment, and proceeded to drop a *C*=*O* to form a *m/z* 117 fragment (Fig. S1A, see online supplementary material). Through a similar process, we qualitatively identified various phenolic acyl groups, such as cinnamoyl, coumaroyl, caffeoyl, and feruloyl (Table 1). Finally, 3Q-MS in MRM mode was utilized to confirm and targeted detect the identified compounds, with the same RT shared in different fragments which were generated from the same precursors taken as the final identified phenolamides. The MRM conditions were then optimized, and the entire workflow is illustrated in Fig. 2.

**Table 1:** The information of identified phenolamides in tea plant

**Peak/no.**	**RT (min)**	**[M + H]** ^**+**^ **(*m/z*)**	***m/z* of main fragments (MS/MS)**	**Identification**	**Abbreviations**	**Acylation degree**
PA01–1	3.155	261.17151	131.04947, 114.10314, 103.05484, 72.08167, 202.12311	*N*-CinnamoylAgmatine	Cin-Agm	Single
PA01–2	3.796	261.17151	131.04947, 114.10314, 103.05484, 72.08167, 202.12311	*N*-CinnamoylAgmatine	Cin-Agm	Single
PA02	2.478	277.16623	147.04433, 119.04963, 72.08172, 93.07064, 111.08103	*N*-*p*-CoumaroylAgmatine	Cou-Agm	Single
PA03–1	5.459	439.23105	147.04416, 123.04446, 204.10233, 163.03911, 72.08166	*N*-*p*-Coumaroyl-*N*″-CaffeoylAgmatine	Cou-Caf-Agm	Twice
PA03–2	5.542	439.23105	147.04416, 123.04446, 204.10233, 163.03911, 72.08166	*N*-*p*-Coumaroyl-*N*″-CaffeoylAgmatine	Cou-Caf-Agm	Twice
PA04	4.502	453.34128	147.04431, 289.061, 177.05508, 119.04967, 72.0817	*N*-*p*-Coumaroyl-*N*″-FeruloylAgmatine	Cou-Fer-Agm	Twice
PA05–1	2.776	219.14964	131.04948, 202.12291, 103.05486, 72.08169	*N*-Cinnamoylputrescine	Cin-Put	Single
PA05–2	3.377	219.14970	131.04945, 202.12285, 103.05484, 72.0816	*N*-Cinnamoylputrescine	Cin-Put	Single
PA05–3	4.857	219.14975	131.04948, 72.08167, 202.12292, 103.05486, 89.1081	*N*-Cinnamoylputrescine	Cin-Put	Single
PA06–1	2.128	235.14453	147.0443, 72.08169, 119.04968, 89.10813, 218.11807	*N*-*p*-Coumaroylputrescine	Cou-Put	Single
PA06–2	2.34	235.14453	147.0443, 72.08169, 119.04968, 89.10813, 218.11807	*N*-*p*-Coumaroylputrescine	Cou-Put	Single
PA07–1	2.332	265.15518	177.05493, 145.02861, 72.08168, 117.034, 89.10812	*N*-Feruloylputrescine	Fer-Put	Single
PA07–2	2.568	265.15518	177.05493, 145.02861, 72.08168, 117.034, 89.10812	*N*-Feruloylputrescine	Fer-Put	Single
PA08–1	2.596	339.14487	147.04427, 177.05498, 327.63724, 145.02864, 119.04965	*N*-Benzoyl-*N*″-*p*-Coumaroylputrescine	Ben-Cou-Put	Twice
PA08–2	3.076	339.14487	147.04427, 177.05498, 327.63724, 145.02864, 119.04965	*N*-Benzoyl-*N*″-*p*-Coumaroylputrescine	Ben-Cou-Put	Twice
PA08–3	3.396	339.14487	147.04427, 177.05498, 327.63724, 145.02864, 119.04965	*N*-Benzoyl-*N*″-*p*-Coumaroylputrescine	Ben-Cou-Put	Twice
PA09–1	4.429	381.13922	147.0443, 352.23914, 235.14445, 119.04967, 72.0817	*N*′, *N*″-Di-Coumaroylputrescine	Di-Cou-Put	Twice
PA09–2	4.662	381.13922	147.0443, 352.23914, 235.14445, 119.04967, 72.0817	*N*′, *N*″-Di-Coumaroylputrescine	Di-Cou-Put	Twice
PA09–3	4.815	381.13922	147.0443, 352.23914, 235.14445, 119.04967, 72.0817	*N*′, *N*″-Di-Coumaroylputrescine	Di-Cou-Put	Twice
PA10	4.343	397.17444	147.04425, 235.14439, 163.0392, 251.1394, 72.08168	*N*-Caffeoyl-*N*″-*p*-Coumaroylputrescine	Caf-Cou-Put	Twice
PA11–1	4.729	411.19641	121.02883, 139.03912, 177.05489, 147.04427, 235.14474	*N*-Feruloyl-*N*″-*p*-Coumaroylputrescine	Fer-Cou-Put	Twice
PA11–2	4.875	411.19641	121.02883, 139.03912, 177.05489, 147.04427, 235.14474	*N*-Feruloyl-*N*″-*p*-Coumaroylputrescine	Fer-Cou-Put	Twice
PA11–3	4.989	411.19641	121.02883, 139.03912, 177.05489, 147.04427, 235.14474	*N*-Feruloyl-*N*″-*p*-Coumaroylputrescine	Fer-Cou-Put	Twice
PA12–1	3.36	276.18466	131.04945, 202.12297, 232.18108, 112.1125, 72.08167	*N*-CinnamoylSpermidine	Cin-Spd	Single
PA12–2	3.961	276.20743	131.04942, 202.12296, 259.18097, 112.11269, 72.0816	*N*-CinnamoylSpermidine	Cin-Spd	Single
PA13–1	2.806	292.20230	147.04433, 119.04967, 72.08173, 204.10233, 275.17569	*N*-*p*-CoumaroylSpermidine	Cou-Spd	Single
PA13–2	3.154	292.20230	147.04433, 119.04967, 72.08173, 204.10233, 275.17569	*N*-*p*-CoumaroylSpermidine	Cou-Spd	Single
PA14–1	5.257	438.23941	147.0443, 204.10226, 119.04967, 72.0817, 292.20221	*N*′, *N*″-Di-*p*-CoumaroylSpermidine	Di-Cou-Spd	Twice
PA14–2	5.446	438.23950	147.04428, 204.10217, 119.04965, 292.20212, 72.08157	*N*′, *N*″-Di-*p*-CoumaroylSpermidine	Di-Cou-Spd	Twice
PA14–3	5.533	438.23999	147.04428, 139.0392, 204.10233, 163.03931, 72.08157	*N*′, *N*″-Di-*p*-CoumaroylSpermidine	Di-Cou-Spd	Twice
PA14–4	5.621	438.24142	147.04427, 163.03929, 123.04451, 204.10216, 72.08167	*N*′, *N*″-Di-*p*-CoumaroylSpermidine	Di-Cou-Spd	Twice
PA15–1	3.361	454.23401	341.09128, 147.04422, 163.0392, 204.1022, 72.08165	*N*-*p*-Coumaroyl-*N*″-CaffeoylSpermidine	Cou-Caf-Spd	Twice
PA15–2	3.475	454.23401	147.0442, 163.03914, 204.10214, 220.09706, 72.08166	*N*-*p*-Coumaroyl-*N*″-CaffeoylSpermidine	Cou-Caf-Spd	Twice
PA15–3	4.956	454.23428	147.04427, 163.0392, 220.09711, 204.1022, 72.08167	*N*-*p*-Coumaroyl-*N*″-CaffeoylSpermidine	Cou-Caf-Spd	Twice
PA15–4	5.13	454.23428	147.04427, 163.0392, 220.09711, 204.1022, 72.08167	*N*-*p*-Coumaroyl-*N*″-CaffeoylSpermidine	Cou-Caf-Spd	Twice
PA15–5	5.217	454.23428	147.04427, 163.0392, 220.09711, 204.1022, 72.08167	*N*-*p*-Coumaroyl-*N*″-CaffeoylSpermidine	Cou-Caf-Spd	Twice
PA15–6	5.312	454.23428	147.04427, 163.0392, 220.09711, 204.1022, 72.08167	*N*-*p*-Coumaroyl-*N*″-CaffeoylSpermidine	Cou-Caf-Spd	Twice
PA16–1	3.783	468.25031	177.05499, 147.04431, 305.05591, 117.03407, 72.08171	*N*-Feruloyl-*N*″-*p*-CoumaroylSpermidine	Fer-Cou-Spd	Twice
PA16–2	3.897	468.25031	177.05501, 305.05569, 165.05507, 72.08168, 204.10228	*N*-Feruloyl-*N*″-*p*-CoumaroylSpermidine	Fer-Cou-Spd	Twice
PA16–3	5.613	468.25031	177.05499, 147.04431, 145.02869, 305.05591, 72.08171	*N*-Feruloyl-*N*″-*p*-CoumaroylSpermidine	Fer-Cou-Spd	Twice
PA17–1	3.473	484.24536	139.0392, 177.05496, 145.02864, 163.03926, 234.1127	*N*-Caffeoyl-*N*″-FeruloylSpermidine	Caf-Fer-Spd	Twice
PA17–2	3.57	484.24536	139.0392, 177.05496, 145.02864, 163.03926, 234.1127	*N*-Caffeoyl-*N*″-FeruloylSpermidine	Caf-Fer-Spd	Twice
PA18–1	5.444	584.27527	147.04402, 438.23889, 204.10194, 72.08157, 420.22821	tricoumaroyl spermidine	Tri-Cou-Spd	Triple
PA18–2	5.538	584.27527	147.04402, 438.23889, 204.10194, 72.08157, 420.22821	tricoumaroyl spermidine	Tri-Cou-Spd	Triple
PA19–1	5.473	615.28058	147.04427, 177.05498, 204.10223, 438.23956, 72.0817	*N*′, *N*″-Di-*p*-Coumaroyl-*N*″'-FeruloySpermidine	Cou-Fer-Spd	Twice
PA19–2	5.626	615.28058	147.04427, 177.05498, 204.10223, 438.23956, 72.0817	*N*′, *N*″-Di-*p*-Coumaroyl-*N*″'-FeruloySpermidine	Cou-Fer-Spd	Twice

As a result, 19 types of phenolamides and their 27 isomers were identified and listed in Table 1, including 14 once-acylated and 32 multiple-acylated (twice or more) modified phenolamides. The secondary mass spectra of all these compounds have been documented (Fig. S2, see online supplementary material). Aliphatic phenolamides, rather than aromatic phenolamides, were mainly accumulated in tea plants and the additive pattern was protonated dominantly.

### Accumulation profile of phenolamides in various organs in tea

**Figure 3:**
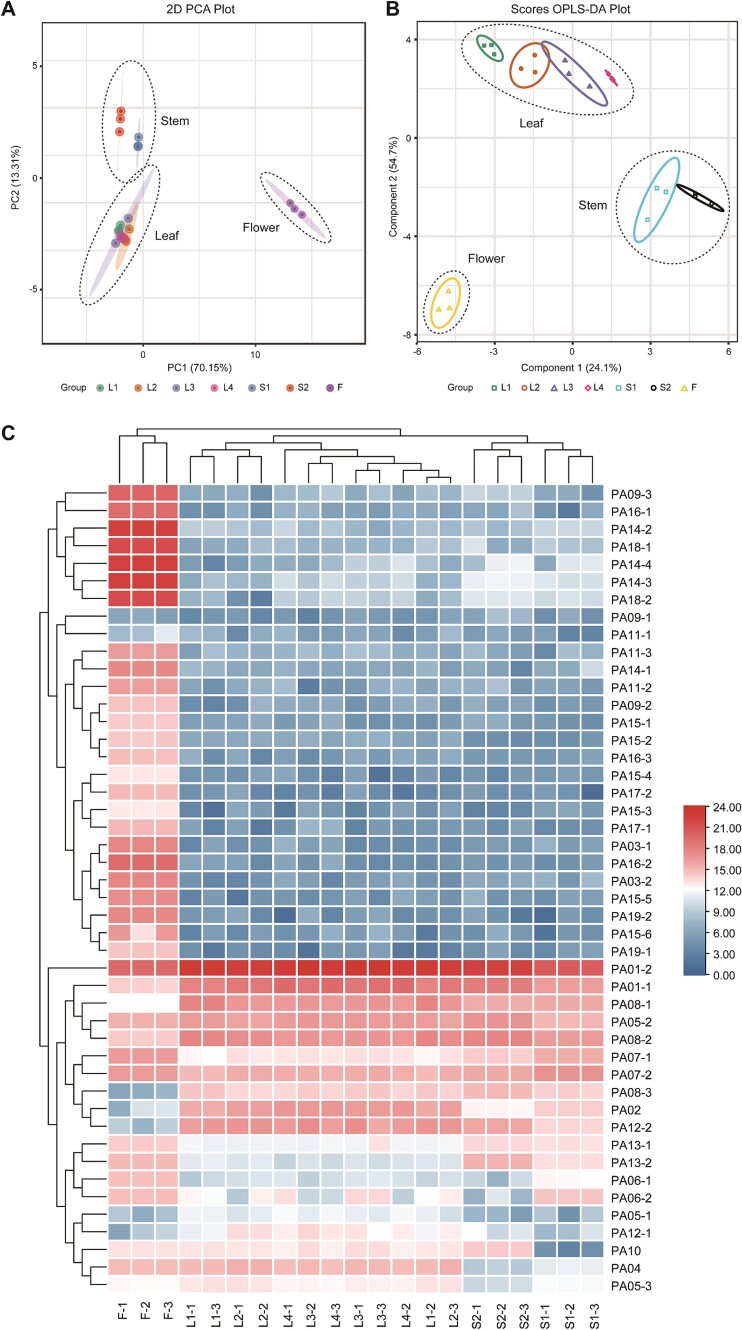
Differential chemotype among various organs of tea, including first leaf (L1), second leaf (L2), third leaf (L3), fourth leaf (L4), stem 1 (S1), stem 2 (S2), and flower (F). **A** and **B** represent PCA and OPLS-DA analysis of phenolamides content identified from seven tea tissues, respectively. **C** HCA analysis of phenolamides identified from seven tea tissues.

In an effort to gain a deeper insight into the accumulation mechanism of phenolamides in tea plants, we conducted a relatively quantitative analysis of phenolamides in different tissues and organs of tea shoots, encompassing first leaf, second leaf, third leaf, fourth leaf, stem 1 (between 1–2 leaves), stem 2 (between 3–4 leaves), and flower organs. After obtaining the semi-quantitative data, a principal component analysis (PCA) was conducted on the entire dataset to understand the metabolic differences and variations among various tissues and organs. The results revealed notable discrepancies in the accumulation of metabolites across various tissues and organs of the tea plant. Principal component 1 accounted for 70.15% of the data set interpretation (Fig. 3A), indicating a strong influence on the results. Flowers, stem 1, and stem 2 were distinguishable from leaves, whereas phenolamide accumulation in leaves of each group was indistinguishable. Although principal component analysis is effective in extracting main information, it lacks sensitivity towards variables with small correlation. Therefore, we opted for orthogonal partial least squares discriminant analysis (OPLS-DA) on the samples. The modeling results indicate a stable and reliable model with a Q^2^ value of 0.953, R^2^Y value of 0.993, R^2^X value of 0.94, and a *P*-value less than 0.005 (Fig. S3, see online supplementary material). The findings of the OPLS-DA analysis were in agreement with those of the PCA analysis, demonstrating that the phenolamides present in tea have the ability to differentiate flowers, stems, and leaves effectively (Fig. 3B).

In order to present a summary of the variations in phenolamides levels across various organs, a hierarchical clustering analysis (HCA) was conducted with the phenolamides content (Fig. 3C; Table S2, see online supplementary material). In accordance with the aforementioned findings, the phenolamides present in flowers, stems, and leaves can be categorized into four distinct groups. Notably, PA09-(2–3), PA14-(1–4), PA15-(1–6), PA17-(1–2), PA18-(1–2), and PA19-(1–2) exhibit significant accumulation in flowers, while their presence in leaves is minimal. PA01- (1–2), PA02, and PA08- (1–3) were found to be present in high concentrations in leaves and stems; however, some isomers displayed different accumulation patterns. For instance, PA05–2 was abundant in all organs, whereas PA05–1 was generally scarce and PA05–3 was mainly observed in leaves (Fig. 3C). It is interesting that the majority of phenolamides that accumulate in flowers are those that have undergone multiple acylation modifications, whereas the phenolamides that have undergone single acylation modifications are mainly accumulated in leaves, such as PA01, PA02, PA05, and PA12 (Table 1 and Fig. 3C). As to the polyamine moiety (the acyl-acceptor), phenolamides in leaves are characterized by the presence of acylation modification forms of agmatine, putrescine, and spermidine, whereas flowers predominantly contain phenolamides with spermidine as the dominant acyl receptor. In addition to the different types and degrees of acyl-modification, the diversity of phenolamides can also be ascribed to the presence of isomers.

### The phenolamides accumulation profile by anthracnose pathogen induction

**Figure 4:**
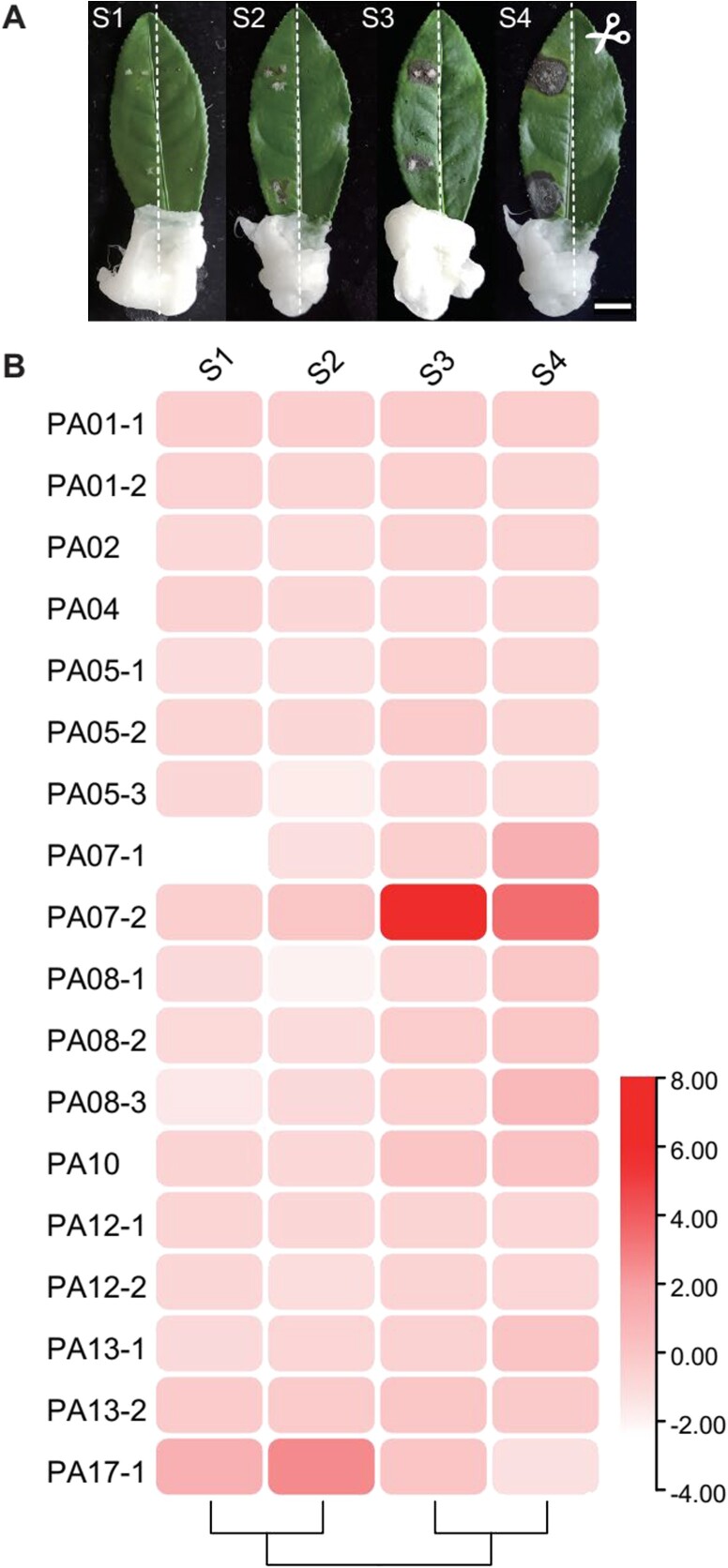
**A** Different stage of diseased tea leaves infected by anthracnose pathogen (*Colletotrichum camelliae*). Bar = 1 cm. **B** The HCA analysis of phenolamides content in tea leaves at diseased stage 1–4.

The leaves of tea possess economic value, and the accumulation of phenolamides in these leaves has prompted concerns regarding their biological significance. The physiological function of phenolamides in disease resistance has been demonstrated in previous research conducted on various plants, with anthracnose being a significant disease that affects tea gardens in China. Thus, we want to explore whether phenolamides are involved in the resistance against anthracnose in tea plants. To investigate this matter, we introduced anthracnose pathogen (*Colletotrichum camelliae*) on one side of the same leaf as the treatment and inoculated the blank culture medium on the other side as the control (Fig. 4A). This aim was to observe the changes of the content of phenolamides in tea leaves at different stages of disease (Table S3, see online supplementary material). The OPLS-DA analysis revealed a clear distinction between the treatment and control groups, particularly in the S3 and S4 stages (Fig. S4, see online supplementary material). Further analysis of the Log2 value of phenolamide relative content changes at each stage showed that the pattern of phenolamide accumulation was similar in the S1 and S2 stages, as well as in the S3 and S4 stages (Fig. 4B). Additionally, with the progression of the disease, there was a decline in the majority of phenolamides present in the leaves. Nevertheless, the presence of certain phenolamides was significantly stimulated by pathogenic bacteria, specifically PA07–2 and PA17–1 (Fig. 4B). Despite an initial increase in the content of PA17–1 during pathogen induction, its overall content remained relatively low. Conversely, the content of PA07–2 (Fer-Put) was abundant in tea leaves and exhibited significant induction with the progression of disease severity, particularly in the S3 stage. Our findings revealed that Fer-Put was significantly induced in response to the pathogen in the susceptible variety ‘*Longjing 43*’ (Fig. 4), as well as in the resistant varieties ‘*Zhongcha 108*’ (Fig. S5, see online supplementary material) and ‘*Shaancha 1*’ (Fig. S6, see online supplementary material), with a corresponding increase in disease severity. The implication is that Fer-Put could be a crucial phenolamide, contributing to the tea plant’s resistance against anthracnose.

### Quantification of Fer-put and its anti-anthracnose evaluation

**Figure 5:**
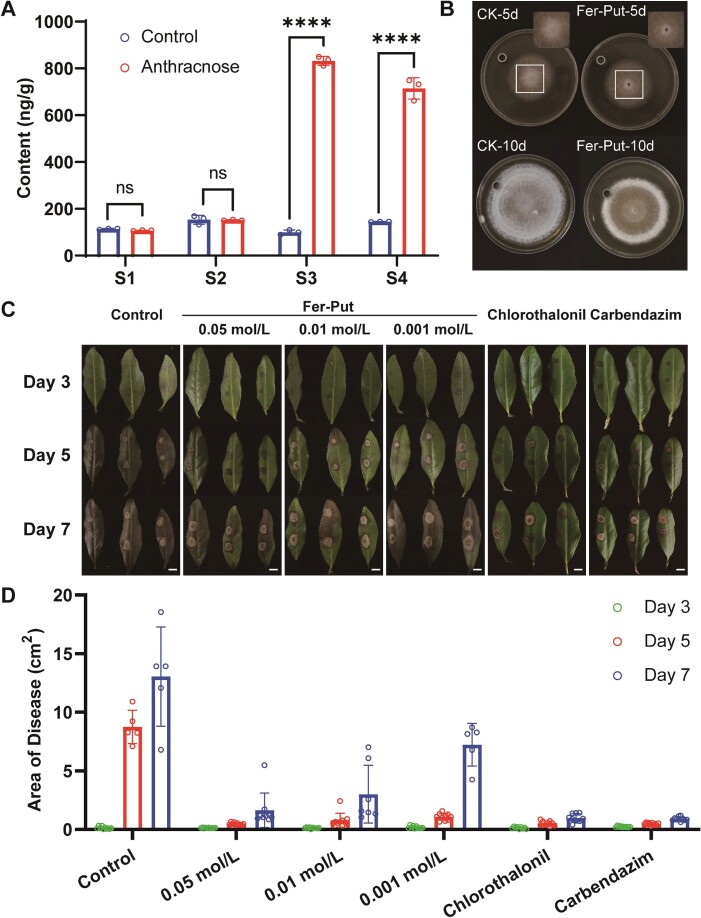
The evaluation of anti-anthracnose function about Fer-Put. **A** The absolute quantitative analysis of Fer-Put content at different stage in diseased tea leaves. **B** The growth of *Colletotrichum camelliae* colony after 5 days and 10 days treated by Oxford Cup, within 100 μl of Fer-Put solution (1 mg/ml) or blank solvent (CK). **C** and **D** indicate the images and the diseased area of tea leaves treated by blank solvent, Fer-Put solution (0.05 mol/L, 0.01 mol/L, and 0.001 mol/L), chlorothalonil and carbendazim after 3 days, 5 days, and 7 days, respectively. Bar = 1 cm in **C**.

To investigate the anti-anthracnose function of Fer-Put in tea leaves, an absolute quantitative analysis of its content in diseased leaves was measured. The findings revealed that the accumulation of Fer-Put in tea leaves increased substantially with the severity of the disease, particularly in the S3 stage where its average content reached to 832.13 ng/g, 8.37 times higher than the control (Fig. 5A). This suggests that the accumulation of Fer-Put is induced by the anthracnose pathogen and may participate in the biological process of anthracnose resistance in tea leaves. Then, we analysed the content of Fer-Put between the susceptible variety ‘*Longjing 43*’ and resistant variety ‘*Zhongcha 108*’. The results showed that the content of Fer-Put increased with the maturity of leaves in both varieties (Fig. S7, see online supplementary material). This is consistent with the resistance to anthracnose of the development degree of leaves, as the pathogen is more likely to infect young leaves. Furthermore, the content of Fer-Put in first, second and third leaves of the resistant variety ‘*Zhongcha 108*’ was significantly higher than that of the susceptible variety ‘*Longjing 43*’, further indicating that Fer-Put could be an important compound in resistance to anthracnose (Fig. S7, see online supplementary material).

To validate its anti-anthracnose function, an Oxford Cup bacteriostasis test was conducted to assess whether Fer-Put can inhibit the growth of pathogen *in vitro*. The results demonstrated a significant reduction in the growth of anthracnose pathogen after exogenous addition of Fer-Put, indicating its antifungal function *in vitro* (Fig. 5B). Then, tea leaves were selected as the subject of research to assess the disease-resistant properties of Fer-Put. The experiments involved treating the tea tree surfaces with varying concentrations of Fer-Put (0.05 mol/L, 0.01 mol/L, and 0.001 mol/L), with blank solvent as the negative control. It is very common and effective to use chlorothalonil and carbendazim to control tea anthracnose in production, so they are used as positive controls (75% chlorothalonil and 50% carbendazim wettable powder 1000 times solution) in this study to assess the disease resistance effect of Fer-Put. The results indicated that Fer-Put significantly reduced the diseased area of tea leaves compared to the blank control, although its effect was not as strong as that of Chlorothalonil and Carbendazim after seven days (Fig. 5C and D). This result was also confirmed on two disease-resistant varieties, ‘*Zhongcha 108*’ (Fig. S8A and B, see online supplementary material) and ‘*Shaancha 1*’ (Fig. S8C and D, see online supplementary material). These findings suggest that Fer-Put is induced and accumulated by the anthracic pathogen in tea plants and possesses biological activity in resisting anthracnose.

### The activities of antioxidant enzymes after Fer-put treatment

**Figure 6:**
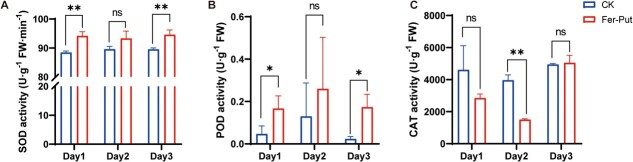
The antioxidant enzymes’ activities of tea leaves after treated by Fer-Put or blank solvent (CK) in threedays. **A**, **B** and **C** showed the SOD activity, POD activity and CAT activity, respectively.

To further explore the anti-anthracnose function of Fer-Put, the activities of antioxidant enzymes were measured in tea leaves. It was observed that the activities of superoxide dismutase (SOD) and peroxidase (POD) were significantly increased after treatment with Fer-Put on the first and third days, while the catalase (CAT) did not show a notable increase (Fig. 6). These results showed that applying Fer-Put could enhance the activity of SOD and POD of tea leaves, suggesting that the anti-anthracnose capability of Fer-Put may be attributed to the stimulation of antioxidant enzymes.

### The correlation between the Fer-put content and the acyltransferase expression level during the pathogenesis

**Figure 7:**
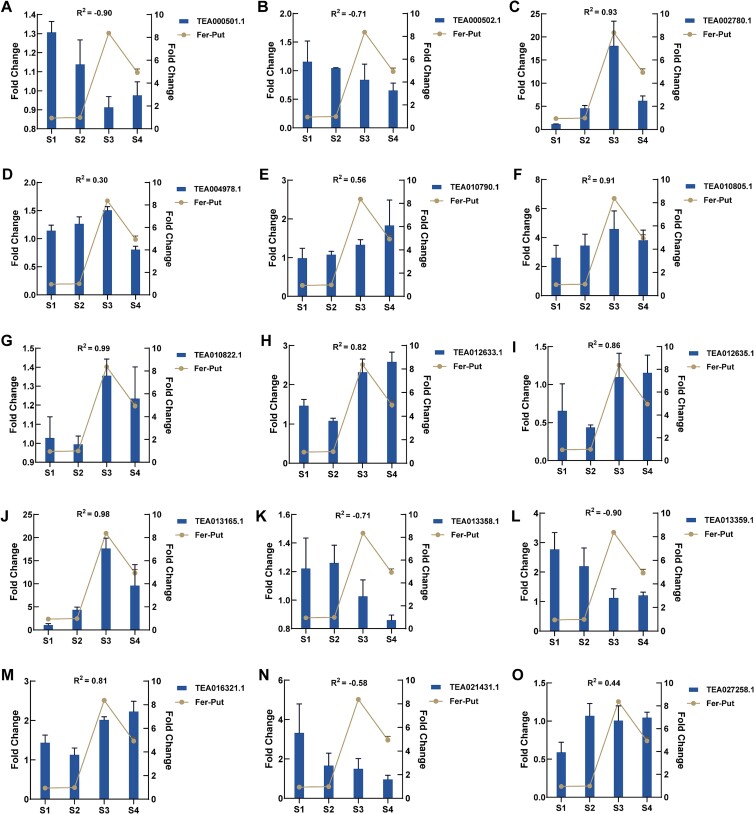
The correlation the Fer-Put content and the acyltransferase expression level during the pathogenesis, **A**–**O** indicated the genes of TEA000501.1, TEA000502.1, TEA002780.1, TEA004978.1, TEA010790.1, TEA010805.1, TEA010822.1, TEA012633.1, TEA012635.1, TEA013165.1, TEA013358.1, TEA013359.1, TEA016321.1, TEA021431.1 and TEA027258.1, respectively.

With the increase of disease severity in tea leaves, the amount of Fer-Put also increases, suggesting that the acyltransferase responsible for the biosynthesis of Fer-Put in tea plants may have a similar expression pattern. Therefore, a correlation was analysed between the Fer-Put content and the acyltransferase expression level during the pathogenesis. As the acyltransferases have the specificity towards polyamine substrate, the functional genes in rice (*OsPHT* and *OsPHT3*) [21] and tobacco (*NaAT1*) [22] that could catalyze the putrescine were used to blast in tea genome for screening the candidate genes. At least 15 genes were identified as potential acyltransferases, and their primers for qPCR were optimized and listed in Table S4 (see online supplementary material). The expression levels of these genes were analysed in the tea leaves infected with anthracnose. Results indicated that the expression of *TEA002780.1*, *TEA010805.1*, and *TEA013165.1* were significantly enhanced in the infected tea leaves, particularly at stage 3 and 4, and their expression levels were strongly correlated with the accumulation of Fer-Put (Fig. 7). Notably, the fold change of *TEA002780.1* and *TEA013165.1* were as high as 18.07 and 17.64 at stage 3, respectively. Additionally, the expression levels of *TEA012633.1*, *TEA012635.1*, and *TEA016321.1* are also positively associated with an increase in Fer Put content, whereas *TEA000501.1* and *TEA013359.1* had a negative correlation (Fig. 7). These results suggest that these genes may play a key role in the biosynthesis of Fer-Put during the pathogenesis, especially *TEA002780.1* and *TEA013165.1*.

## Discussion

Phenolamides, a class of secondary metabolites, are widely present throughout the plant kingdom. The existence of various acyl receptors and donors, along with an array of modification sites, contributes to the abundance of phenolamides in plants. Moreover, the lack of commercial standards for phenolamides poses a challenge to their identification using mass spectrometry. With the advancement of technological methods, there has been a consistent analysis of the various types and structures of phenolamides found in different plants. For instance, the 31 phenolamides and their 33 *cis*/*trans* isomers were identifed in 20 types of monofloral bee pollen, and their MS/MS cleavage modes were summarized as well [23]. Tea plants possess a rich variety of secondary metabolites, but the identification and analysis of phenolamides in tea have not been reported systematically so far. In this study, we employed UHPC-Orbitrap-MS/MS and UPLC-QQQ-MS/MS techniques to develop a workflow for the identification of unknown metabolites with accurate mass in different tissues and organs of tea plants. Our study is the first to comprehensively and systematically identify and analyze phenolamides in tea plants, including their structures (including isomers) and accumulation patterns. This workflow for identifying metabolites is based on high-resolution orbitrap mass spectrometry, which enables the collection of high-quality signals in wide mass ranges. Subsequently, the candidate metabolites are obtained by screening and comparing with the high-resolution mass spectrometry data with theoretical value predicted from speculated compound. Each candidate metabolite is then verified using 3Q-MS, and 3–10 ion pairs are selected for MRM detection. The presence of at least three ion pairs peaking at the same retention time confirms the target compound. The MRM detection conditions of the target compounds are optimized to generate the final detection method. This metabolites identification workflow is not only applicable to the identification of phenolamides but also to other widely targeted metabolites, particularly those with specific structural modifications.

During the metabolic analysis, it was discovered that tea plants predominantly accumulate aliphatic phenolamides, rather than aromatic phenolamides. In addition, our results indicate that the buildup of phenolamides in tea plants is specific to certain tissues and acyl-acceptors, and this distribution is associated with the extent of phenolamide modification. Several investigations have demonstrated that the acyltransferases involved in the biosynthesis of phenolamides displays a preference for particular acyl receptors, specifically polyamines. To date, there have been two types of identified acyltransferases in plants capable of catalyzing the formation of phenolamides [24, 25]. These enzymes include tyrosine: *N*-hydroxycinnamoyl transferases (THTs), which are classified under the *GNAT* family, and the *BAHD* family (BEAT, AHCT, HCBT, and DAT), which respectively correspond to the benzylalcohol O-acetyl transferase, anthocyanin O-hydroxycinnamoyl transferase, anthranilate N-hydroxycinnamoyl/ benzoyl transferase, and deacetylvindoline 4-O-acetyl transferase [26]. Aromatic phenolamide, particularly tyramine, is generally considered the optimal substrate for THT [27]. It is worth noting that aliphatic phenolamides tend to accumulate in tea plants. As a result, it is our hypothesis that the biosynthesis of these compounds is primarily catalyzed by the BAHD acyltransferases. Numerous studies have demonstrated the multifaceted functions of BAHD proteins. These proteins, located in distinct evolutionary branches, exhibit selectivity or varying catalytic activities towards diverse polyamine substrates. For instance, the *Hv*ACT in barley [3] and *Os*AHT1 in rice [21] clustered in clade IV, have the ability to facilitate the production of phenolamide from agmatine. Meanwhile, *Sr*SpmHT of *Solanum richardii* [28] in clade III can utilize spermine as a polyamine substrate. In this research, we examined the acyltransferases in tea plants by utilizing *OsPHT*, *OsPHT3*, and *NaAT1*, which have the capacity to catalyze the biosynthesis of putrescine. Additionally, we analysed the association between the expression levels of candidate genes in different stages of disease and the amount of Fer-Put, uncovering the key genes that could be responsible for Fer-Put biosynthesis in the anthracnose resistance process. Exploring whether the key genes in tea plants are responsible for biosynthesize of Fer-Put will be a crucial area of focus for our future investments.

Although the structure and distribution of phenolamide have been thoroughly documented [8, 9, 29, 30], their physiological or biological roles remain poorly comprehended [31–33]. It is noticed that a plenty of phenolamides of diverse varieties have been accumulated in the reproductive structures of several plant species, including those of tea plants. Yet, the specific purpose behind their abundant presence in floral organs and pollen envelopes remains unclear [10, 34, 35]. Some research suggests that the presence of phenolamides in flowers may be associated with plant reproduction, such as the involvement in floral initiation and flower fertility. Furthermore, they are also believed to be a vital component of the pollen coat in eudicotyledons [36, 37].

Numerous studies have demonstrated that phenolamides possess protective functions against both biotic and abiotic stressors, such as pathogens [5, 38, 39], insects [33], mineral deficiencies [40, 41], high temperatures [42], wounding [43], and more. Fer-Put is suggested as it may play an essential role in stress resistance. In 1970, researchers discovered that wheat infected with stripe rust had increased levels of Fer-Put and Cou-Put (p-coumaroylputrescine) [44]. These two compounds have been proven to respond to the attack of the brown planthopper on plants in rice [21, 33], and the acyltransferases that synthesize them in rice leaves can be significantly induced by wounding expression [21], suggesting that they may be involved in the stress process of resistance to mechanical damage. In addition, Fer-Put and aromatic phenolamides (benzoyltryptamine and benzoylserotonin) levels increases significantly in leaves affected with the pathogens *Xanthomonas oryzae* pv. *oryzae* (*Xoo*) or *Cochliobolus miyabeanus* [45], indicating that Fer-Put may be an important disease-resistant compound. It has also been demonstrated that Fer-Put is involved in the growth and development process of plants, as it is a marker of cortical callus formation in all explants of *Nicotiana tabacum* [46], and can respond to the stimulation accumulation of *Azospirillum* in rice roots to improve plant growth and health [47]. The protective role of phenolamides against microbes involves at least two processes: direct antimicrobial activity and reinforcement of the secondary cell wall [2]. As to the first process, it found that tyramine- and tryptamine-based phenolamides have been found to possess growth inhibition properties against *Xanthomonas sp*. [45, 48], while p-coumaroylnoradrenaline, p-coumaroyldopamine, and feruloyldopamine are highly effective on suppressing the growth of *Pseudomonas syringae* [5, 31]. The present study has demonstrated the significant inhibitory effect of Fer-Put on the colony growth of the tea anthracnose pathogen, as well as its ability to reduce the infection rate of tea leaves. It was also discovered that the application of Fer-Put to tea leaves can significantly enhance their antioxidant enzyme activities, such as SOD and POD, which may be a factor contributing to the increased resistance of tea trees to anthracnose. These findings represent the first report on the resistance function of phenolamides in tea plants, and the first report on the involvement of phenolamides in the resistance to plant anthracnose. However, further research is required to determine whether phenolamides play a role in reinforcing the secondary cell wall upon pathogenic infection.

In summary, this research establishes a research foundation for the development of natural small molecules sourced from tea plants that exhibit inherent immunity against anthracnose, along with the precise selection of resistant cultivars. It also establishes a theoretical framework for the environmentally sustainable prevention and control of tea gardens.

## Material and methods

### Plant materials, pathogen isolate, pathogenesis, and disease resistance experiments on tea leaves

In the current investigation, the susceptible variety *Camellia sinensis* ‘*Longjing43*’ [49] cutting seedlings were employed to detect phenolamides levels in various tissues and to perform pathogenetic experiment on tea leaf. As an addition, the pathogenetic experiments were also conducted with the use of the resistant variety *C. sinensis* ‘*Shaancha1*’ [50] and *C. sinensis* ‘*Zhongcha108*’ [49]. The pathogen responsible for tea anthracnose was isolated from diseased tea leaves at Xixiang Tea Experimental Demonstration Station of Northwest Agricultural and Forestry University in Shaanxi Province, China. Following isolation, purification, and DNA identification, it was determined to be a strain of *Colletotrichum camelliae*, a known pathogen of tea anthracnose.

Experiments were conducted using healthy tea leaves of comparable size and developmental stage. Symmetrical wounds were created on each leaf using a sterile needle, and a 5 mm mycelial plug of the pathogen was then inoculated on the wound site at the back of the leaf. For 48 hours, the leaves were cultivated in a controlled environment of 25°C and 70% relative humidity. Observations were made at four different time points (1d, 3d, 5d, and 7d) and the area of the lesions was measured by ImageJ software (1.53 t, National Institutes of Health, USA). For disease resistance experiments on tea leaves, Fer-Put, chlorothalonil, and carbendazim were sprayed in specific concentrations on the surface of tea leaves for 3 days. Afterwards, the leaves were inoculated with pathogen cake and the area of lesions was then measured.

### Chemicals and solvents

Sigma-Aldrich Chemical Co. (St Louis, MO, USA) supplied LC–MS grade acetonitrile (ACN) and methanol (MeOH). The standard products *N*-Feruloylputrescine (CAS No. 501–13-3) and *N*-*p*-Cumaroyltyramine (CAS No. 36417–86-4) were procured from Shanghai Bairen Biotechnology Co., Ltd (Shanghai, China) and ChemFaces (Wuhan, Hubei, China), respectively. 75% chlorothalonil and 50% carbendazim wettable powder were purchased from Shandong Libang Agrochemical Co., Ltd (Qingdao, Shandong, China) and Zhenjiang Jiansu Pesticide Chemical Co., respectively.

### Extraction of metabolites

Plant materials were collected and immediately frozen in liquid nitrogen, then stored at −80°C. The freeze-dried samples were then pulverized in a mixer mill (MM 400, Retsch) with zirconia beads for 1 minute at 30 Hz. An approximate mass of 80–120 mg of the powder was extracted with 640–960 μl of 70% aqueous methanol (v/v) in an ultrasonic ice bath for 10 minutes. After centrifugation at 10000 *g* for 10 minutes at 4°C, the extracts were filtered using a SCAA-104 filter with a 0.22 mm pore size (ANPEL, Shanghai, China). The remaining residue was extracted twice using the same process. The two supernatants were then combined for LC–MS analysis.

### Liquid chromatography-mass spectrometry

For the high-resolution mass spectrometry analysis part, a Dionex UltiMate 3000 UHPLC system (Thermo Fisher Scientific) was utilized for LC, with water and acetonitrile (each containing 0.04% acetic acid) as phases A and B, respectively. Reverse phase separation of analytes was then conducted on a Kinetex XB-C18 column (100 × 2.1 mm, 2.6 μm particle size; Phenomenex). The column oven was maintained at a temperature of 40°C during the analysis of tea samples using a gradient of 5–95% B for 20 minutes, 95% B for 2 minutes, 95–5% B for 0.1 minute, and maintaining 5% B for 2.9 minutes, with a flow rate of 0.35 ml min^−1^ and an injection volume of 2 μl. An interface of UPLC and a Thermo Scientific™ Exactive™ Plus hybrid quadrupole-Orbitrap mass spectrometer was established using a heated electrospray ionization (HESI-II) interface. The mass spectrometer was configured to work in both positive and negative ionization mode, and had a full scan range of 100–1500 m/z and the top ten data-dependent MS/MS scans. The parameters set were spray voltage 3.5 kV, capillary temperature 350°C, sheath gas 40 psi, aux gas 10 psi, probe heater temperature 350°C and S-lens RF level 55.

For the QQQ-MS/MS analysis part, it was performed on 1290 InfinityII-6470 UPLC system (Agilent Technologies, Santa Clara, CA, USA). Chromatographic separation of phenolamides was performed on an ACQUITY UPLC HSS T3 column (100 Å, 1.8 μm, 2.1 mm X 100 mm, 1/pkg; Waters, Milford, MA, USA) with column temperature maintained at 40°C. Binary mobile phases with phase A of water containing 0.04% acetic acid (v/v) and phase B of acetonitrile were containing 0.04% acetic acid (v/v) used for elution, respectively. The linear gradient program was as follows: 0 min, 5% B; 12 min, 95% B; 13.2 min, 95% B; 13.3 min, 5% B; 15 min, 5% B. The flow rate was 0.35 ml/min and the injection volume was 1 μl. To enhance the detection conditions, the capillary outlet voltage (fragmentor) was optimized using Agilent.

MassHunter Workstation Optimizer 10.1 in the SIM mode to ensure efficient transmission of the product ion fragment. Then, two product ions with high intensity have been selected for qualitative and quantitative ion analysis. The dwell time with longer quantitative ion distribution was chosen for the final optimized detection conditions of each compound.

### Anti-anthracnose test by Oxford cup experiment

In order to evaluate the efficacy of Fer-Put against anthracnose, a fungal cake of *Colletotrichum camelliae* measuring 5 mm in diameter was introduced at the center of a petri dish containing PDA medium. Subsequently, a sterilized and dried Oxford Cup was carefully inserted into the upper layer of the petri dish using sterile tweezers. Then, an Oxford Cup was placed equidistantly from the center of each culture dish. The 100 μl of Fer-Put solution with a mass concentration of 1 mg/ml was injected into the Oxford Cup using a sterile microinjector, serving as the experimental group. Meanwhile, the control group was administered with a blank solvent. The culture dishes were placed in an artificial incubator, which was set to 25°C and 70% relative humidity in a dark environment. The growth of the colony was monitored and recorded.

### The determination of antioxidant enzymes’ activity

The UV-1800 spectrophotometer (Shimadzu, Japan) was used to determine the activity of superoxide dismutase (SOD), peroxidase (POD) and catalase (CAT) according to a previously described protocol [51]. In brief, the samples for the assessment of antioxidant enzymes, SOD, POD, and CAT were treated with nitrogen blue tetrazole, guaiacol, and hydrogen peroxide methods, respectively.

### Statistical analysis

The principal component analysis (PCA) and opted for orthogonal partial least squares discriminant analysis (OPLS-DA) were performed on https://cloud.metware.cn/. The heatmap was constructed by TBtools software (v1.098769). Statistical analyses were performed using GraphPad Prism 9. By conducting an analysis of *t*-test, the means and standard deviations of the data were determined and statistically analysed.

## Acknowledgements

We thank Miss Jing Zhao (Horticulture Science Research Center, Northwest A&F University, Yangling, China) for providing professional technical assistance with LC–MS/MS analysis. Some text in this paper was polished by Stork's Writing Assistant (https://www.storkapp.me/writeassistant/). This work was supported by the Natural Science Foundation of China (32202551), the Natural Science Basic Research Program of Shaanxi (2022JQ-194), the earmarked fund for Modern Agro-industry Technology Research System (CARS-19), the National Key Research and Development Program (2022YFD1602000), the Agricultural Special Fund Project of Shaanxi Province (NYKJ-2022-YL(XN)37) and the special fund for University-Supported Extension Model (TGZX2022-2).

## Author contributions

W.W. and Y.Y. conceived and designed the experiments. W.W., X.X., H.G., L.B., C.G. and J.Z. collected the tea samples. W.W., Y.L., X.X., L.L., Q.H., H.G., and Y.B. performed the experiment. W.W. and X.X. analysed the data and performed the visualization. W.W. drafted the manuscript. All authors have read and approved the manuscript.

## Data availability

All data supporting the conclusions of this study may be found in the publication and its supplemental materials, which are available online. Any additional relevant information can be obtained from the corresponding author upon request.

## Conflict of interest statement

The authors declare no conflict of interest.

## Supplementary data

Supplementary data is available at *Horticulture Research* online.

## Supplementary Material

Web_Material_uhad154
