# Supplementary material for: Identification and profile of phenolamides with anthracnose resistance potential in tea (Camellia sinensis)
Source: Hortic Res. 2023 Aug 1;10(9):uhad154. doi: 10.1093/hr/uhad154 (PMC10500153; doi:10.1093/hr/uhad154)
Supplement: Web_Material_uhad154 [file web_material_uhad154.zip › Supplementary figures-updated.pptx]

## Slide 1
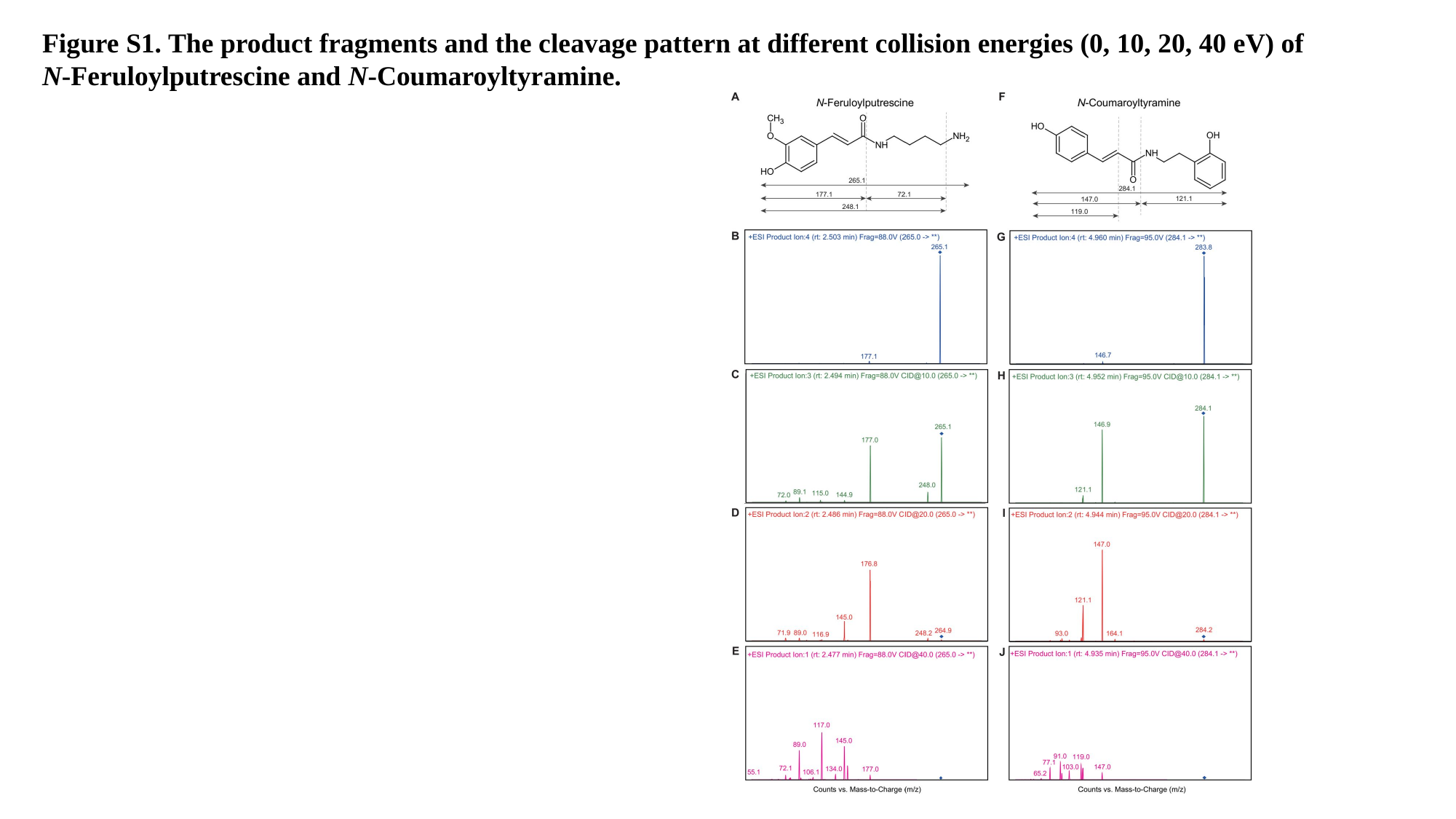

Figure S1. The product fragments and the cleavage pattern at different collision energies (0, 10, 20, 40 eV) of N-Feruloylputrescine and N-Coumaroyltyramine.

## Slide 2
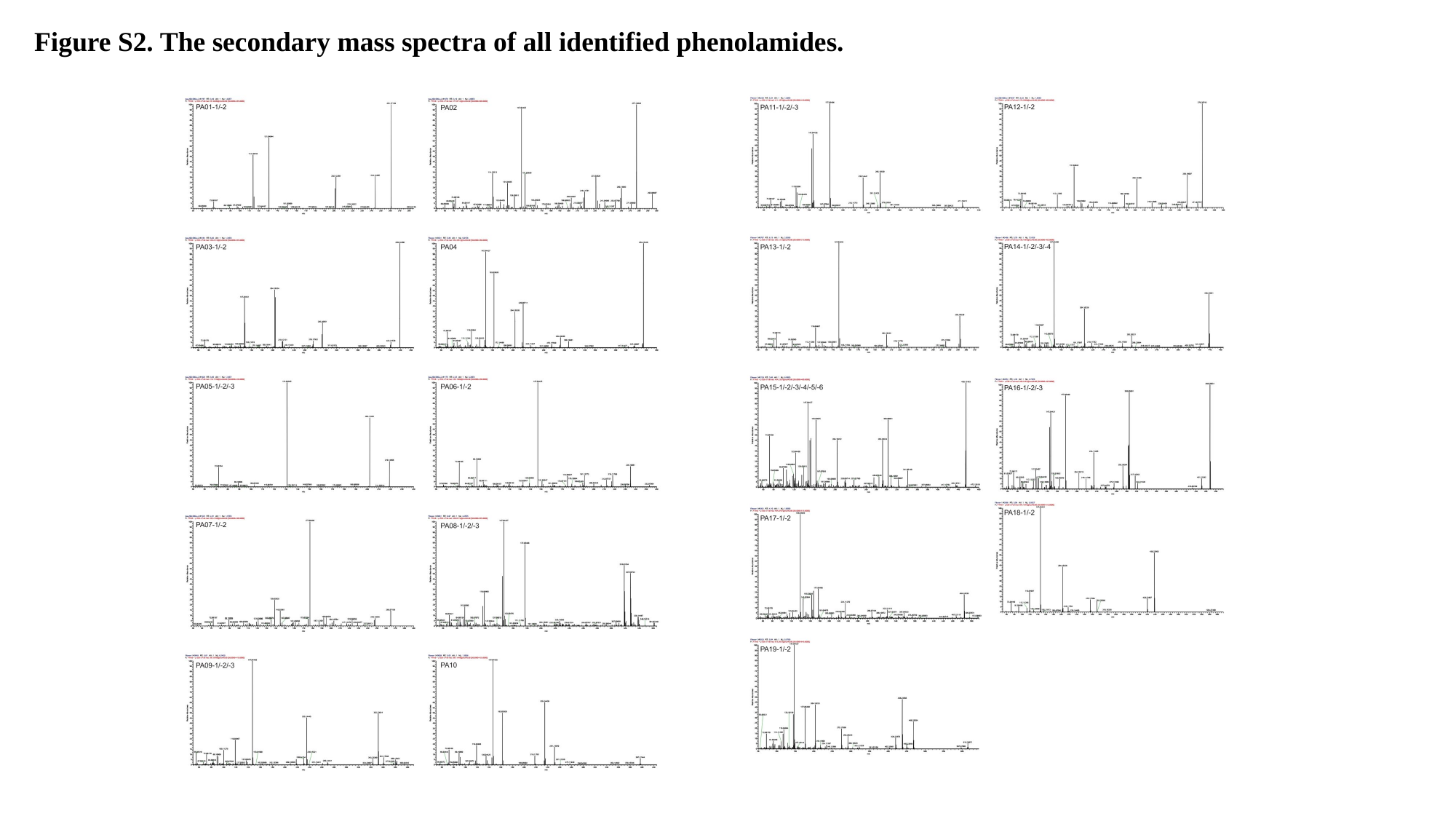

Figure S2. The secondary mass spectra of all identified phenolamides.

## Slide 3
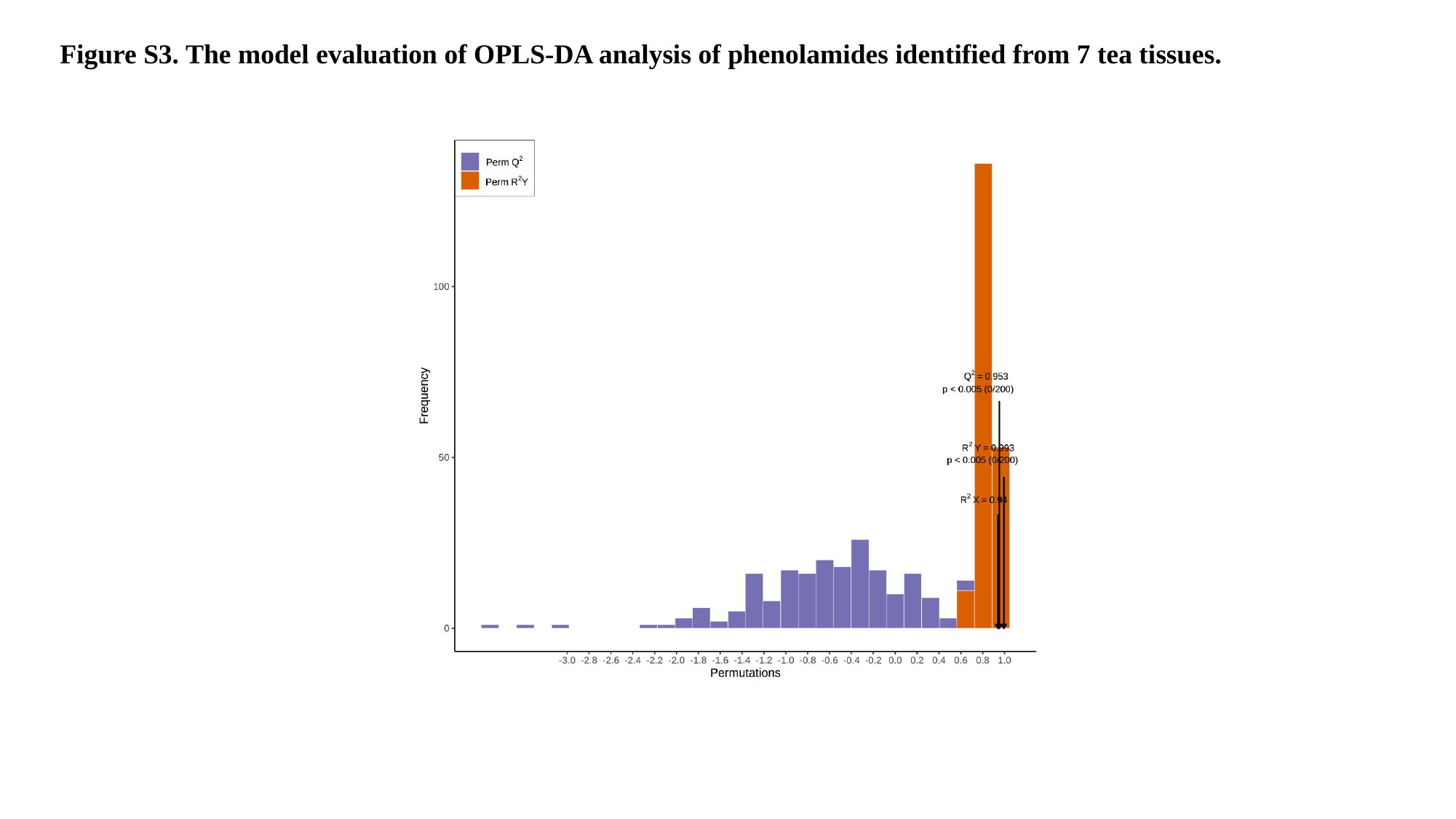

Figure S3. The model evaluation of OPLS-DA analysis of phenolamides identified from 7 tea tissues.

## Slide 4
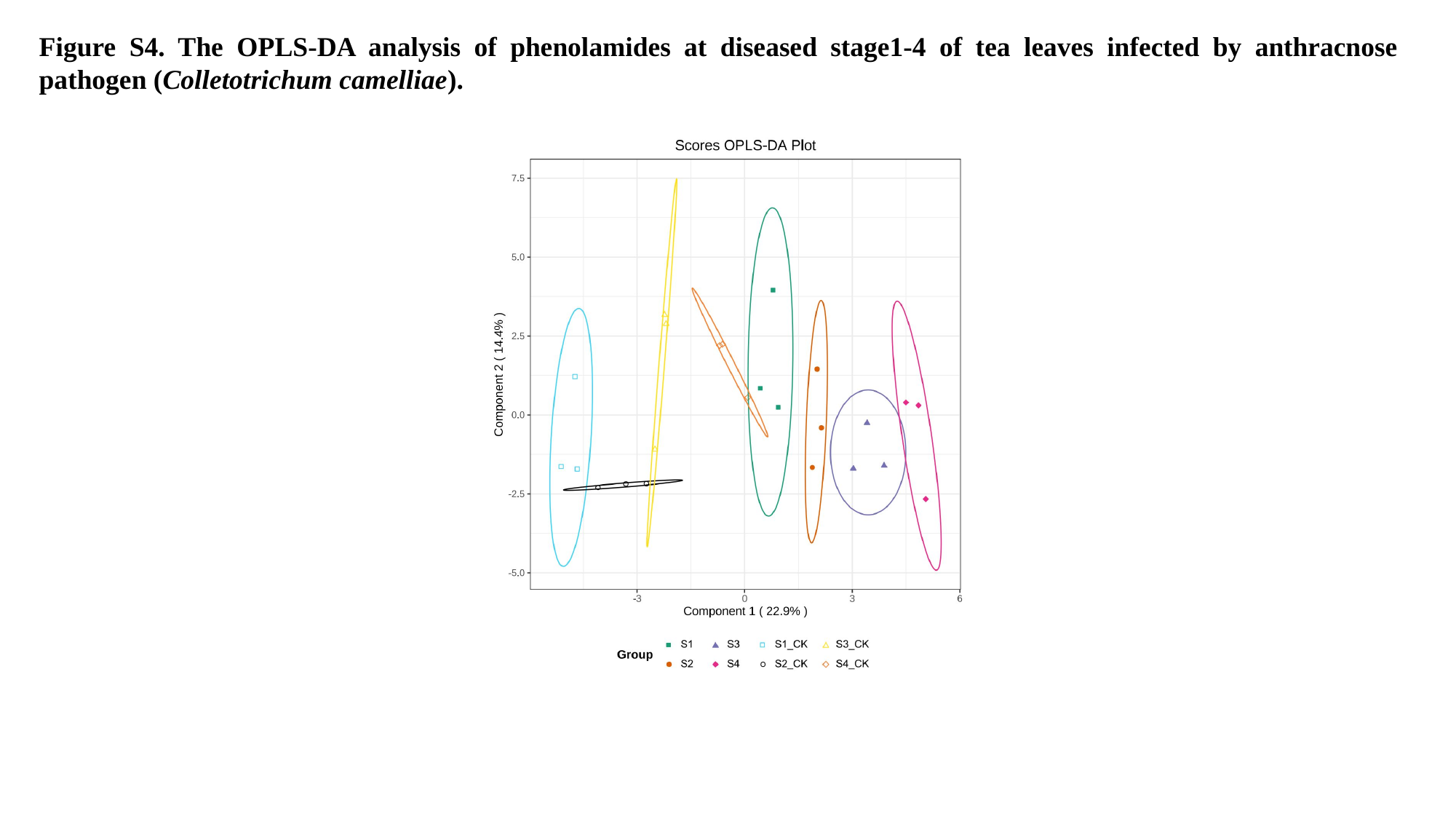

Figure S4. The OPLS-DA analysis of phenolamides at diseased stage1-4 of tea leaves infected by anthracnose pathogen (Colletotrichum camelliae).

## Slide 5
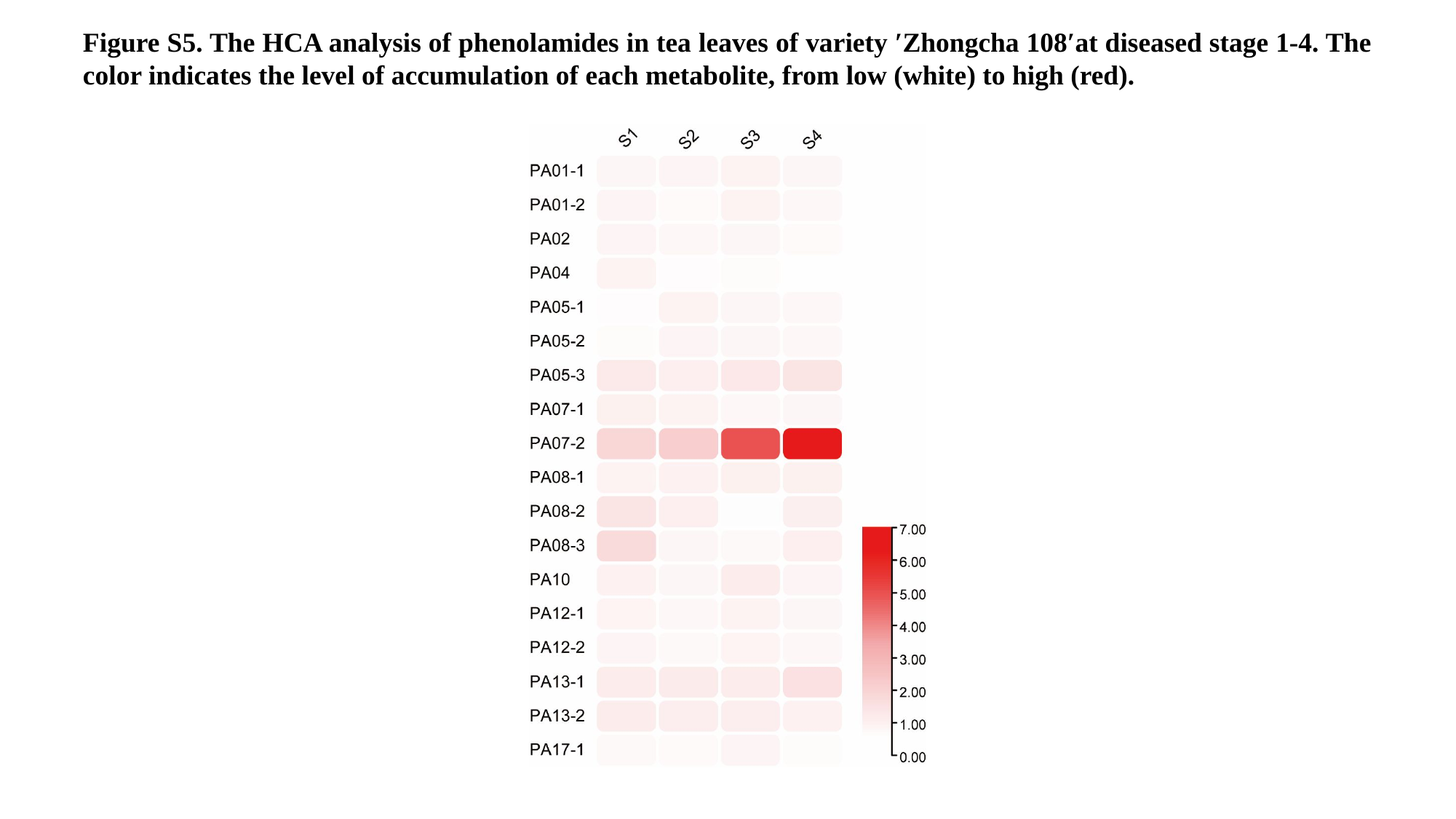

Figure S5. The HCA analysis of phenolamides in tea leaves of variety ′Zhongcha 108′at diseased stage 1-4. The color indicates the level of accumulation of each metabolite, from low (white) to high (red).

## Slide 6
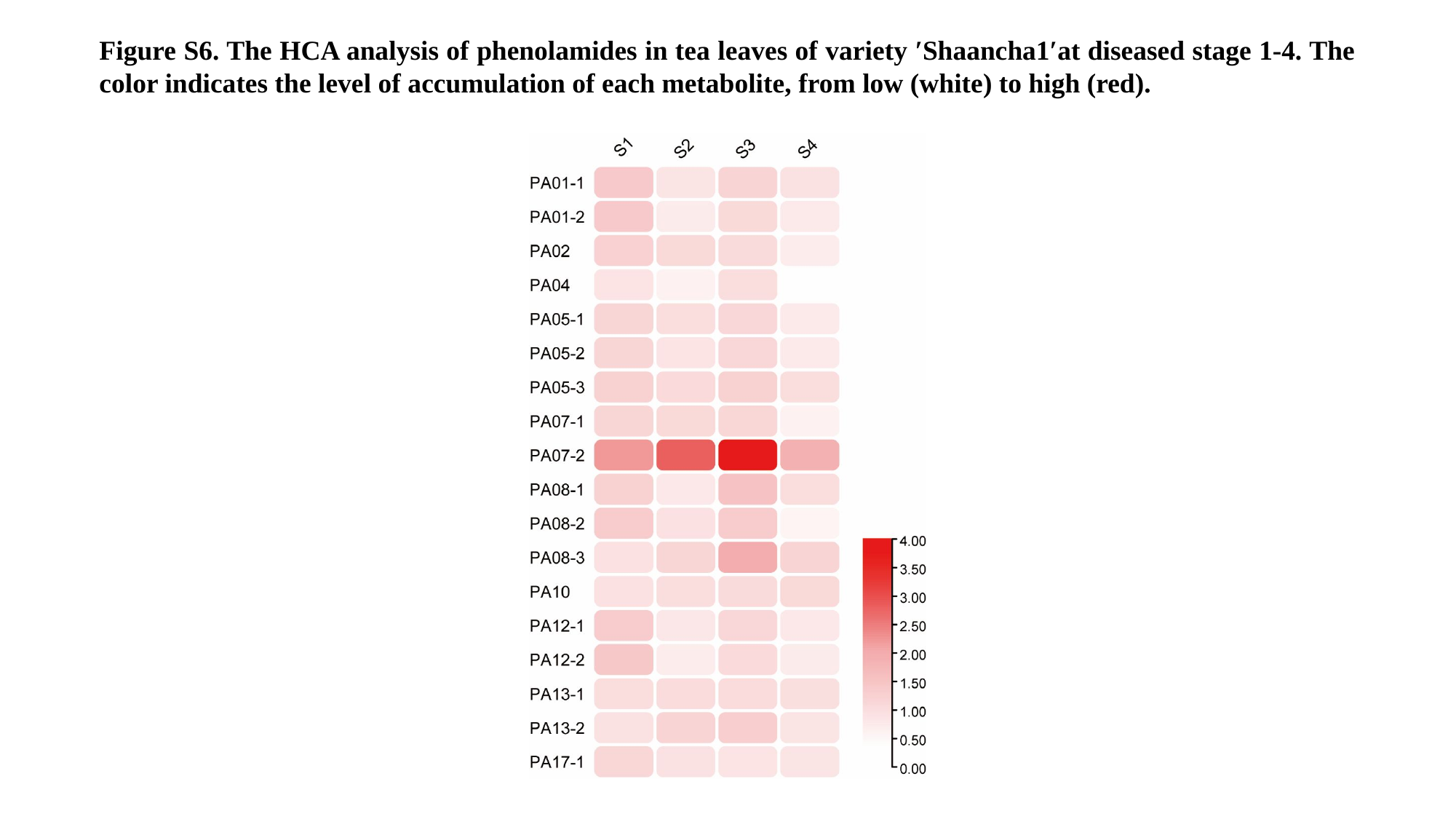

Figure S6. The HCA analysis of phenolamides in tea leaves of variety ′Shaancha1′at diseased stage 1-4. The color indicates the level of accumulation of each metabolite, from low (white) to high (red).

## Slide 7
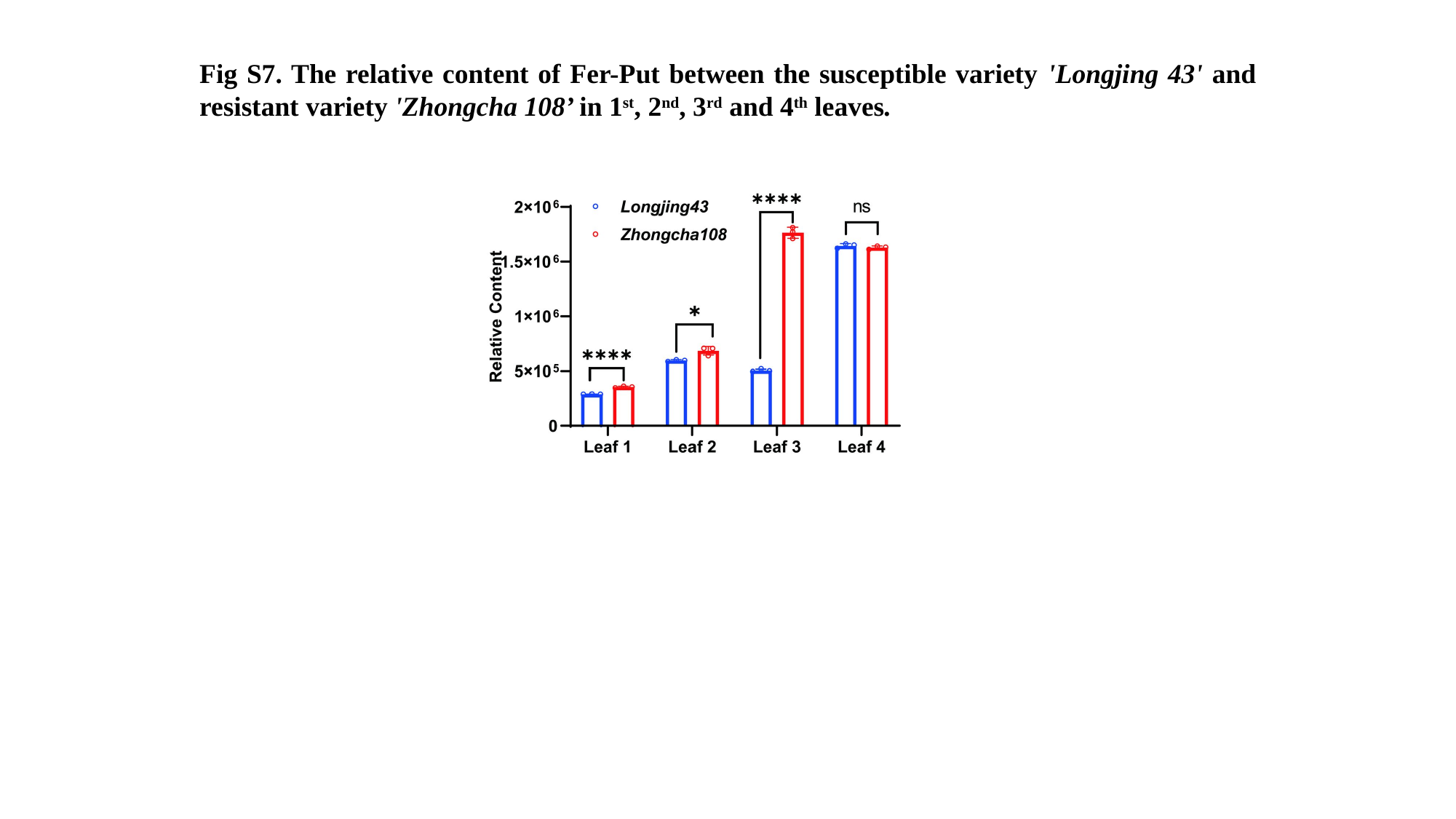

Fig S7. The relative content of Fer-Put between the susceptible variety 'Longjing 43' and resistant variety 'Zhongcha 108’ in 1st, 2nd, 3rd and 4th leaves.

## Slide 8
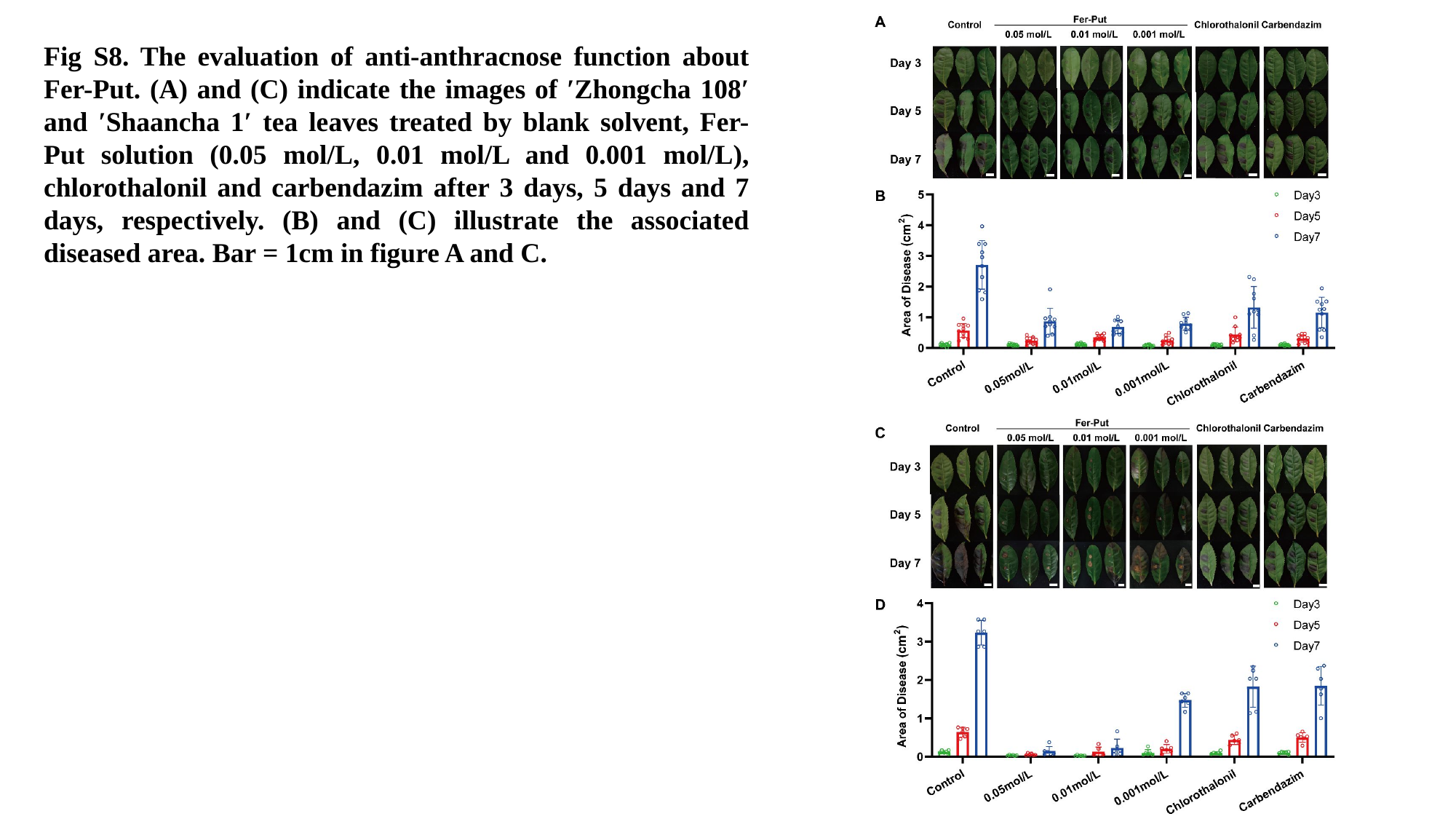

Fig S8. The evaluation of anti-anthracnose function about Fer-Put. (A) and (C) indicate the images of ′Zhongcha 108′ and ′Shaancha 1′ tea leaves treated by blank solvent, Fer-Put solution (0.05 mol/L, 0.01 mol/L and 0.001 mol/L), chlorothalonil and carbendazim after 3 days, 5 days and 7 days, respectively. (B) and (C) illustrate the associated diseased area. Bar = 1cm in figure A and C.
